# The Wooden Skull: An Innovation through the Use of Local Materials and Technology to Promote the Teaching and Learning of Human Anatomy

**DOI:** 10.1155/2020/8036737

**Published:** 2020-08-27

**Authors:** Kintu Mugagga, Masilili G. Mwarisi, Samuel S. Dare

**Affiliations:** ^1^Department of Human Anatomy, Faculty of Biomedical Sciences, Kampala International University, Western Campus, Uganda; ^2^Department of Anatomy, Faculty of Medicine, Mbarara University of Science and Technology, Uganda; ^3^Department of Human Anatomy, School of Health Sciences, Makerere University, Uganda; ^4^Kabale University School of Medicine, Kabale University, Uganda

## Abstract

Skeleton models are important in facilitating a student's easy retention and recollection of information in the future. These may assist students carry out hands-on practice in order to acquire and practice new skills that are relevant to first aid. The increasing number of medical institutions and medical students attracts the challenge of inadequate facilitation of the teaching and learning processes. This warrants a study and/or an exploration of an alternative solution such as wooden models in order to solve the problem of scarce and ethically restricted human teaching aids. Wooden pieces (50 cm length × 20 cm diameter) from a *Jacaranda mimosifolia* tree were prepared for the carving process, and wooden replicas of human skulls were made. Two experimental groups of randomly selected medical students (60: active and 60: control) were separately taught using wooden and natural skull models, respectively. The two groups were assessed and evaluated using the natural skull models to compare their understanding of the anatomy of the skull. Additionally, opinion statements were collected from participants in the active group during the oral examination. Six (6) wooden skull models were produced and used for experimental study. Comparisons of academic scores (mean and median) between active (students using the wooden skull) and control (students using natural skull) groups showed no statistically significant difference (*P* ≥ 0.05). Concerning the enhancement of learning skills, the wooden model was constructed in a way that would be able to enhance learning as it would be the natural skull. The wooden skull model, with more improvement in structural formation, can adequately facilitate the teaching and learning of anatomy of the human skull. This project and the experimental study about utilization of the wooden skull model provide a good potential of using the wooden models to supplement the use of the natural human skull.

## 1. Introduction

Human skeletal anatomy models, especially human skull anatomy models, are great for patient education and students, in both educational and medical settings. Many classroom environments use skeletal systems to teach their students about the bone structure of the human body especially considering the complexity of structures in the skull. To understand difficult concepts, visual aids such as skeleton models are important in facilitating a student's easy retention and recollection of information in the future. Also, these skeleton models may assist students carry out hands-on practice in order to acquire and practice new skills that are relevant to first aid. In this regard, students in the early years of their medical training can practically demonstrate their ability for thorough and proper assessment to their teachers or examiners [1].

The training of medical doctors in the preclinical years requires many dissections, but legal and ethical issues limit the availability of cadaveric material in many countries. Due to the scarcity of these skeleton materials, students have access to the materials for a limited duration. There is also the challenge of always transmitting unknown or known infectious agents from a cadaveric skull [1].

The use of models for teaching has been reported by several studies including the studying of muscular, cardiovascular, and digestive systems and peripheral nerves by students who dissected cat which were observed to score lower marks than those who sculpted clay models of the body systems [2, 3]. Therefore, it is important to note that the importance of anatomical models as learning tools in understanding complex three-dimensional (3D) anatomical structures cannot be overemphasized.

Over the past 30 years, 3D printing has been used widely globally as described firstly by Charles W. Hull in 1986. 3D technology has been employed to construct several anatomical models such as bones, skull, heart, and kidney for educational purposes [4–7]. Although the skull has always remain the most complicated areas of anatomy, the use of skull base models in endoscopic training and temporal bone anatomy education, for example, is of great importance [8]. According to Chen et al. [9], a randomized control trial (RCT) study designed to compare the learning effectiveness of 3D-printed skulls with cadaveric skulls and the atlas revealed that study effectiveness especially the ability to recognize structures was enhanced better with the use of a 3D-printed skull compared with traditional learning materials.

The increasing number of medical institutions and medical professional students is practically a reality which positively address Medical Educational Partnership Initiative- (MEPI-)Theme No. 1, Increasing the Quantity and Quality of Health Professionals [10] and more globally supporting the MDGs [11, 12]. However, this quickly attracts major challenges particularly the facilitation of the teaching and learning processes. For any concerned teacher in any medical institution, effective and efficient teaching and learning are principally based on student centeredness, activity at classroom level, and individualization amongst others [13]. At present, a major challenge currently faced by Ugandan medical institutions is the large class, often with over 300 biomedical students.

According to Sugand et al. [14], the future of teaching medical student anatomy beyond the next ten years will probably be based largely on independent learning aids. With a ratio of 30 students/skull, this is 4 times less than the standard ratio of students per skull that is generally accepted [15]. In terms of the costs, the price of the human skull is hard to define due to ethical values/restrictions. The hardship involved in acquiring the human model and the ethical rules governing its utilization outweigh any form of pricing system and thus make it hard to attach the real monetary value. Although plastic models have also been adapted, the cost of acquiring them is enormous for institutions in a developing country like Uganda.

Nevertheless, there is a consistent increase in the interest to develop and employ new educational tools for teaching since it has been established that the use of visual aids like models enables students to perform better in anatomy course examination [16].

Comparatively, the wooden model once accurately constructed, the carvists, and the anatomists using their mastery can generate as many models as required at low cost as $100 per wooden skull. With about 12 human skulls available at gross anatomy laboratory at African institutions, this leads to a student/skull ratio of 30 which is far less than the standard ratio accepted.

Thus, this warrants a study and an exploration of an alternative solution to the problem of scarce and ethically restricted human teaching aids.

*J. mimosifolia* is a spectacular tree found in many tropical and subtropical countries. Similar to many other ornamental trees, it is regarded as native to South America; however, it has spread widely over the century, naturalized in many countries, and also penetrated into many locations in East African countries including Uganda [17–19]. *J. mimosifolia* is an invaluable tree having impact economically, socially, and environmentally; as such, it is useful as carvings and tool handles, interior carpentry wood source for fences, and fuel plant [19]. In this regard, we chose *J. mimosifolia* because the timber is yellowish white, moderately heavy, but hard and easy to work with.

## 2. Materials and Methods

### 2.1. The Wood Source: *Jacaranda mimosifolia*

**Figure 1:**
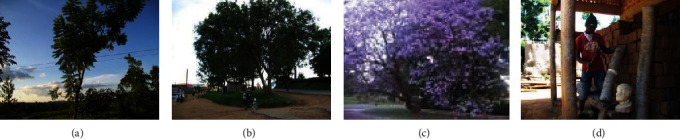
The wood source and cutting into logs.

Building our skull model begins with a wood source, *Jacaranda mimosifolia* (Figure 1) which was harvested from Nyendo Village, Masaka District, Western Uganda. Our choice was based on its physical properties, namely, the yellowish-white color, moderately heavy, fine textured, attractive, close grained, and figured. Additionally, *J. mimosifolia* has good working qualities like lightness and easy to work with, and it stains, glues, and sands easily. Its density is established to be 0.615 g/CC [17–19].

### 2.2. The Wood Processing

**Figure 2:**
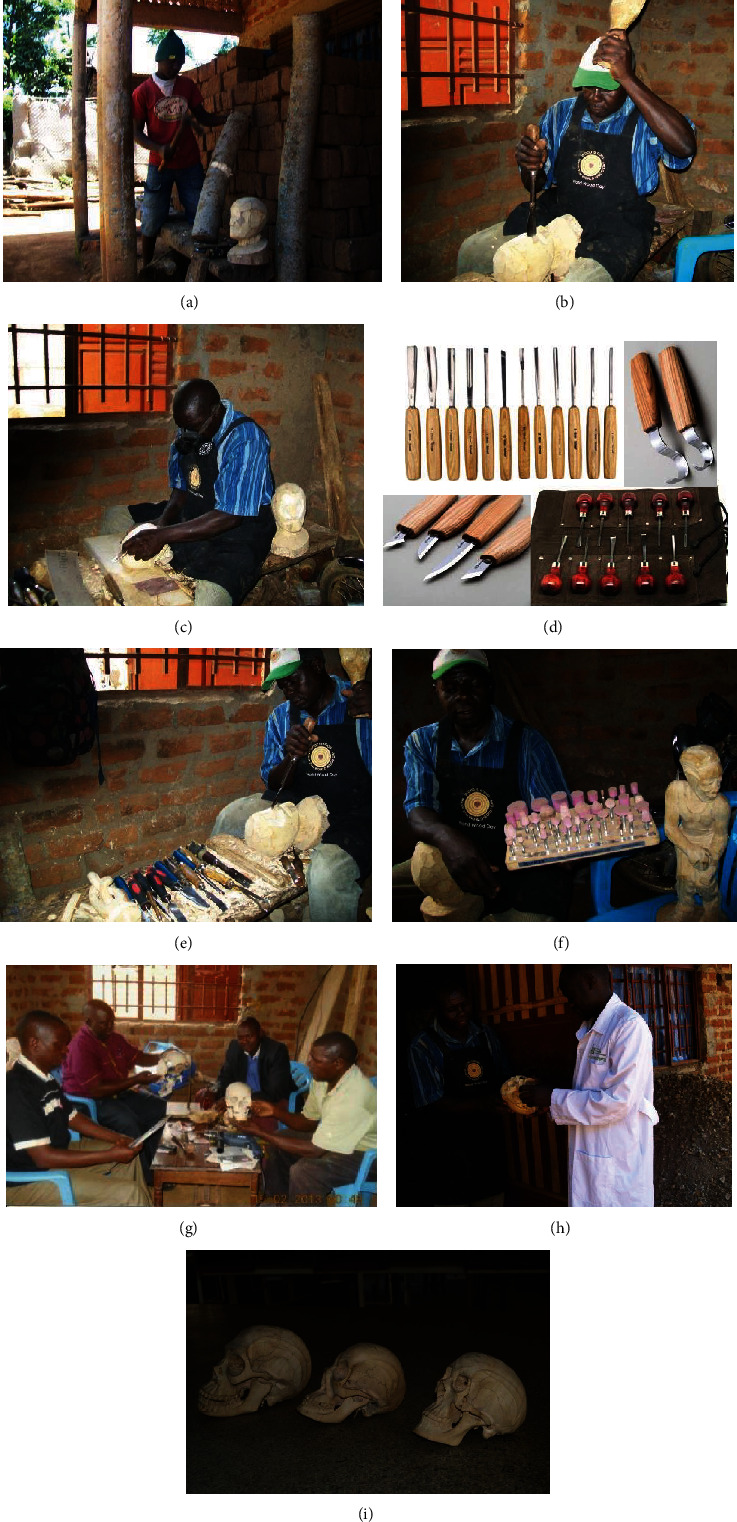
Wood carving process, tools, technical team, and finished product.

We cut with a hand saw 10 cylindrical pieces (50 cm in length and 20 cm in diameter) each from a 10 m Jacaranda tree (mature height). We dried the pieces under shade and debarked them afterwards using Stanley chisels (12-25 mm). After weight reduction, down to approximately 9.5 kg in 30 days, we subjected them to primary coarse-carving (Figures 1 and 2(a)). We carried out the wood processing at the Department of Human Anatomy, Kampala International University, Ishaka Campus, in collaboration with wood carvists of the Kampi Art and Crafts Wood Carving Center Kitovu, Masaka, Uganda.

### 2.3. The Carving Tools

We were guided by the wood carvists, and an assortment of carving tool was bought from different sources in Kampala. However, a good number of them could not be found on the market. Collaboratively, we hired the workshop tools from Kampi carvists under a contractual agreement. The following tools were used: beginner carving tool set, wood carving kit, whittling knife kit, whittling jack, ultimate power sharpener, palm carving tools, micro carving tools, miniature carver, power chisel, angle grinders, brick and mortar saw, round and oval eye punches, carving scraper set, mallet size set, digital calipers, and others (Figures 2(d)–2(f)).

### 2.4. The Carving Process

The proportionate dimensions of the wooden skull model under transformation were based on the anatomical guidelines provided by the medical illustrator (Mr. Paul Lukiza, Makerere College of Health Sciences) (Figures 2(b), 2(c), 2(e), and 2(h)).

### 2.5. The Guiding Models

For perfection, we used the following reference models: (i) an exploded human skull model, (ii) an intact complete human skull, (iii) a hemi- (sagittal) section of the human skull, (iv) a human skull without skull cap (calvaria) exposing the cranial cavity, and (v) human anatomy textbooks [12], showing the elaborate anatomy of the human skull (Figure 2(g)).

### 2.6. The Carving Output

After 8 weeks (including 4 weeks of seasoning period), we successfully produced six (6) wooden skull models (Figures 2(h) and 2(i)). On average, each skull took 1 week of carving. The carved skull models were used for the experimental study with the biomedical science students.

### 2.7. Experimental Study to Validate the Use of Wooden Skull for Anatomy Education

We randomly selected two groups of medical students (60 in each group), from the MBChB Class (year 2-semester 1). The two groups were fully notified about the details and intentions of the experimental study. They were briefed about their rights as participants in the study, and their consent and willingness were sought for according to required guidelines.

We taught the anatomy of the human skull to the two groups separately using wooden and the natural models for the active and control groups, respectively, in using gross anatomy laboratory 1 and gross anatomy laboratory 2, respectively. The teaching took place in May 2013 for 24 hrs in 4 days covering skull development, splanchnocranium, neurocranium, scalp, muscles, and joints. The periods covered theory and practical concerns. The two groups were equally facilitated by two anatomists, i.e., each group had two anatomy lecturers (ratio, 30 students/lecturer) who used similar curricular guidelines to cover the topics. The student/skull ratio was for both groups 10 : 1, and every lecture was followed by a practical session. Due to limitations in time, the carving of the cranial cavity was omitted and concentration of the study and assessment was limited to external anatomical details on the wooden skull model.

### 2.8. Assessment

We comparatively assessed the two groups by subjecting them to a standard exam which had written, practical, and oral sections. The examination was based on the traditional natural human skull model, and proportionally, the practical scored 50%, written 30%, and oral 20%. Additionally, an opinion statement about the wooden skull (as compared to the natural skull) was collected from participants in the active group during the oral examination.

### 2.9. Statistical Analysis

For data analysis using SPSS (version 18), comparative scores of the two groups (from the written, practical, and oral exams) were analyzed using two-sample *t*-test with equal variances. The chi-squared test was used to determine the statistical difference between the two sets of scores. Finally, comparative scores between the two groups showed no significant difference (*P* ≥ 0.05).

A descriptive statistics was employed to broadly evaluate the effect of the treatment group on student perceptions about the activity and science. The students' open-ended responses were qualitatively analyzed using an inductive reasoning where related responses were grouped into subsets that are quantifiable. In this respect, the responses were categorized by the researchers independently and reached agreement of not less than 90% on their categories after further discussion [20].

### 2.10. Ethical Considerations

The study was approved by Mbarara University of Science and Technology (MUST) Institutional Review Board IRB (No. 08/09-12) and recommended for registration with Uganda National Council for Science and Technology.

## 3. Results

### 3.1. Appearance of Cranial Features

**Figure 3:**
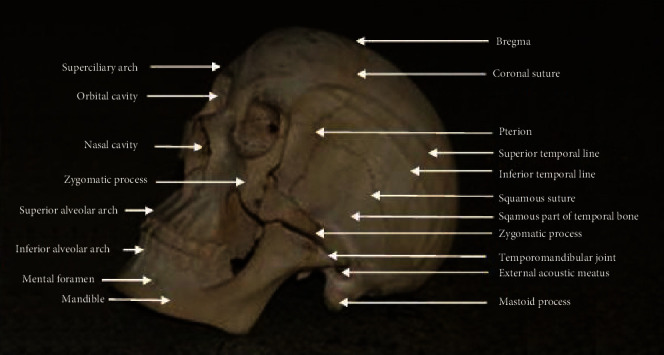
Our wooden skull with some anatomical features labelled.

The appearance of our wooden skull specimen model demonstrated the anatomical features of the natural skull (Figure 3). Most of the bones and sutures such as coronal, squamosal, lambdoid, and parietomastoid were clearly shown. Other features clearly demonstrated are orbital cavity, nasal cavity, pterion, mastoid process, mandible and upper and lower jaws, zygomatic arch, supra- and infratemporal fossa, temporal lines, temporomandibular joint, occipital protuberances, etc.

### 3.2. Null Hypothesis

Teaching the anatomy of the human skull by using an artificial model provides similar exposure and understanding as the cadaveric model.

To test the hypothesis, we compared the group exposed to the human skull with the one exposed to the wooden skull, using scores from written, oral, and practical examination. We set the level of significance 0.05.

### 3.3. Students' Quantitative Evaluation

To understand the failure rate of the students, we categorized the scores as 40-50 (failed), 51-60 (pass), 61-70 (credit), 71-80 (B), and 81-100 (A). We used chi-square to determine the difference.

### 3.4. Students' Qualitative Evaluation

#### 3.4.1. Learner's Reaction, Knowledge Acquisition, Skills, Attitudes, and Behavioural Changes through Converted Oral Exam Transcripts

*(1) Qualitative Findings*. The innovation of a wooden skull model has been presented in 2 perspectives:

- Assessment of the anatomical features of the wooden skull
- Comparison of wooden skull with the natural skull

*(1) Assessment of the Anatomical Features of the Wooden Skull*.

*(1) Theme 1: Fixed Joints*. To innovate a model for anatomical learning experience from wood takes great courage, skills, and knowledge. Framing a skull from wood can equally bring out a structure like that of a natural skull. Though some structures are not easy to carve out exactly as the natural skull, some parts like the joints may be fixed at certain places. According to respondents,

…*The wooden skull has a fixed temporomandibular joint*.

*(2) Theme 2: Clear Demonstration of Sutures*. The sutures were clearly carved out, which may be due to their anatomical makeup which made them easy to carve from wood. This made them clearly identified by the respondents during their learning experience using a wooden skull. According to respondents,

... *Lambdoidal suture and sagittal sutures are clearly demonstrated*.

*(3) Theme 3: Enhancement of Learning Skills*. The wooden model was constructed in a way that would be able to enhance learning as it would be the natural skull. One of the respondents stated:

*Personally I have enjoyed learning using this model ... it has increased my desire to correlate the structures and be able even to know more concerning the true skull*.

*(2) Comparison of Wooden Skull to Natural Skull*.

*(1) Theme 4: Close Connection*. The wooden skull was constructed to have similar structures as the natural skull. This created a close connection and similarity to the natural skull. A respondent revealed that

*there is close connection between the artificial model and the wooden model*.

*(2) Theme 5: Easy to Use for Learning*. Compared to the natural skull, the wooden skull was easy to use in learning. This was due to the simplicity of how it was carved. In this regard, respondents stated:

...*one thing it has caused, is my attraction to the structures of the skull and been able to increase my desire to know more about the skull*.

Also,

*I find the skull model easy to study and learn….*

## 4. Discussion

Supported by Harden and Laidlaw [13] observation in clinical training and assessment where multiple difficulties are encountered in standardizing real patients thus leading to development of simulated patients, the use of natural/human models in quality teaching of human anatomy is characterized by limited choice for (i) the best model, (ii) how many models can be acquired as needed, or (iii) how best to standardize the existing models.

The output of this experimental study about utilization of the wooden skull model clearly signifies the potential of using the wooden models to largely substitute the natural human skull. This is because examination results of our study showed no statistically significant difference between the groups of students exposed to the use of both the natural skull and the wooden skull model (Tables 1–4). Considering the mark distribution of the students' performance, there was no significant difference between the control and active groups in terms of the pass and failure rate (Tables 5 and 6).

According to Ruiz et al. [21], the use of physical learning objects is necessary for robust learning in both preclinical and clinical education and furnishes several research opportunities as well. Embracing learning objects for adaptive learning can easily help to measure the reaction of learners, knowledge acquisition, skills, and attitude as well as behavioural changes. Therefore, transcribing the oral exam of the students as shown in Section *(2) Comparison of Wooden Skull to Natural Skull* of our result revealed no significant difference between the cadaver skull and the wooden skull.

Our observation agreed with the publication of Andreas Vesalius in 1543, *De Humani Corporis Fabrica*, which brought a rebirth in anatomy in Europe as reported by Russell in 1972. This publication highlighted the need for some representation of the body anatomy in the form of image picture and models due to lack of preservative means and scarcity of bodies for dissection. Though the need was partially met by anatomical prints, nevertheless, it was not satisfactory because a single-dimensional picture cannot give the true impression of the body structure as required for an untrained person. Obviously, a three-dimensional figure will be of advantage to a single-dimensional picture despite the fact that it may not reveal details as perfect as it would in the engraving [22].

Khot et al. [23], in an experimental study, compared the results of a knowledge test between learners who studied female pelvic anatomy using a solid 3D plastic model, a static atlas-type compendium of photographic images, and computer-based virtual reality materials. The results revealed that the group of students that used the plastic model remarkably performed better than the other groups which used photographic images and the computer-based virtual materials. Also, Daniel Preece and his mentors Drs. Sarah Williams, Richard Lamb, and Renate Weller in their article reported that students who used a plastic model of an equine foot learnt extremely better than those who used textbooks or 3D computer models [24].

**Figure 4:**
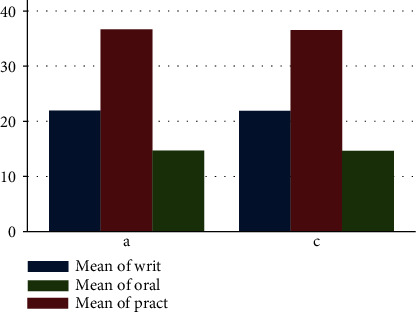
Mean comparison of active (a) and control (c) groups in academic performance. Bar chart showing mean values of student test scores for the written, practical, and oral exams for each treatment (natural skull and wooden skull).

**Figure 5:**
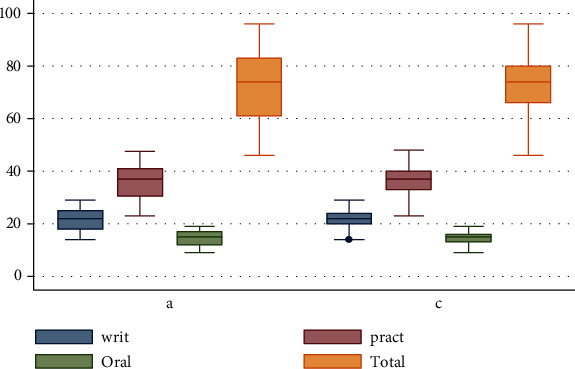
Median comparison of active (a) and control (c) groups in academic performance. Box plot illustrating the median, minimum, and maximum as well as 25–75 percentile ranges of student test scores for the written, practical, and oral exams for each treatment (natural skull and wooden skull).

Our results showing the wooden skull model with the features demonstrated (Figure 3) and that are user-friendly presented a significant impact on the student's attitudes, learning, and retention of the osteology of the skull without statistically significant difference in the mark distribution between the control and active groups (Figures 4 and 5). It is also important to note that the psychological disorder or problems that the natural skull might bring to the students causing them to withdraw from its use and learning well can be solved with the use of the wooden skull model therefore better satisfying the requirement for teaching and learning.

### 4.1. Practical Significance

This wooden skull model shall serve as a starting point in setting up the human anatomy skills lab, which shall improve on availability of teaching models for medical students' teaching and learning. With this production, at least one skull model for every 5 trainees can be achieved, i.e., even a class of 300 learners can ably have 60 skull models available for training. Desired quality teaching and learning practices like small groups, student centeredness, individualization, and activity shall be easily achieved.

### 4.2. Sustainability

#### 4.2.1. Growing Expertise

We have demonstrable evidence that the team that practically participated in the carving process (the wood carvists, medical illustrator, and the anatomists) is now more versed with knowledge and art of fabricating and teaching the anatomy of the skull.

#### 4.2.2. Source of Raw Material

The continued availability of wood being a renewable resource is about growing the tree of choice (*J. mimosifolia* tree) which truly comes along with many other benefits.

#### 4.2.3. Durability of the Wooden Model

Jacaranda wood has a property of durability based on its physical and chemical composition. The models are still treatable with ordinary wood preservative (like Wood-bliss) to prevent microbial (fungal, insect, etc.) degradation under the laboratory storage and usage environment.

#### 4.2.4. Environmental Friendliness (Compatibility with MDG-7)

We cannot ignore the contribution to the green environment as we grow more Jacaranda trees as future source of wood for the models [25]. This is further supported by the high conversion rate (every 10m tree produces twenty 50cm pieces, where each piece is convertible into one wooden skull model, i.e., 20 models per tree). More importantly, the wood carving process is purely mechanical with no chemical involvement (i.e., no addition or emissions of chemical or gases to the environment), contrary to Gregory [26] and contrary to plastics which are manufactured with chemicals that are known to be toxic. In studies in animal model organisms, it was reported that chemicals used in the manufacture of plastics and present in human population have potential adverse health effects [27]. These chemical burdens correlate with adverse health effects such as reproductive abnormalities in the human population [28, 29].

As one of the most naturally renewable energy sources known, wood has less impact on the environment than other materials. It can also last longer than a lifetime when treated correctly, and waste from production of wooden materials is limited and one hundred percent degradable. Given that wooden pallets are more environmentally friendly and more eco-friendly than plastic pallets due to current concerns about climate change [30, 31], this wooden skull model carved from *J. mimosifolia*, though not easy or simple to illustrate all the structures clearly especially the internal structures, may be a better alternative to plastic models. The researcher, therefore, looks forward to overcoming the limitations encountered in the process of carving subsequently by ensuring to improve on the external features as well as properly carving out the internal structures to effectively demonstrate in detail the anatomical structures with minimal defects.

## 5. Conclusion

The outcome of this project and the experimental study about utilization of the wooden skull model at Kampala International University, Anatomy Department, provides a good potential of using the wooden models to supplement the use of the natural human skull. The use of the wooden model for training medical students can be done anywhere and anytime without probable exposure to infection from the cadaver skull and also freedom from ethical and legal restrictions.

## Figures and Tables

**Table 1:** Written exam.

Group	Obs	Mean	Std. Err.	Std. Dev.	(95% Conf. interval)	
Active	50	21.93	0.5350968	3.783706	20.85468	23.00532
Control	42	21.88095	0.5270856	3.415905	20.81648	22.94542
Combined	92	21.90761	0.3754222	3.600923	21.16188	22.65334
diff		0.0490476	0.7578572	-1.456568	1.554663	

*P* = 0.53, *t* = 0.0647, degrees of freedom = 90; based on the above *P* value of 0.53, there is no significant difference in performance of students exposed to human skull with the ones exposed to wooden skull in a written exam. Note that the confidence intervals also overlap while the mean is also the same. Two-sample *t*-test with equal variances.

**Table 2:** Practical exam.

Group	Obs	Mean	Std. Err.	Std. Dev.	(95% Conf. interval)	
Active	50	36.65	0.8742635	6.181977	34.8931	38.4069
Control	42	36.52381	0.8921116	5.781544	34.72215	38.32547
Combined	92	36.59239	0.6224292	5.970131	35.35601	37.82877
diff		0.1261905	1.256444	-2.369956	2.622336	

*P* = 0.54, *t* = 0.1004, degrees of freedom = 90; based on the above *P* value of 0.54, there is no significant difference in performance of students exposed to human skull with the ones exposed to wooden skull in a practical exam. Note that the confidence intervals also overlap while the mean is also the same. Two-sample *t*-test with equal variances.

**Table 3:** Oral exam.

Group	Obs	Mean	Std. Err.	Std. Dev.	(95% Conf. interval)	
Active	50	14.66	0.35209	2.489652	13.95245	15.36755
Control	42	14.61905	0.3507953	2.273413	13.9106	15.32749
Combined	92	14.6413	0.2481809	2.380468	14.14832	15.13429
diff		0.0409524	0.500991	-0.9543536	1.036258	

*P* = 0.53, *t* = 0.0817, degrees of freedom = 90; based on the above *P* value of 0.53, there is no significant difference in performance of students exposed to human skull with the ones exposed to wooden skull in an oral exam. Note that the confidence intervals also overlap while the mean is also the same. Two-sample *t*-test with equal variances.

**Table 4:** Total aggregate score.

Group	Obs	Mean	Std. Err.	Std. Dev.	(95% Conf. interval)	
Active	50	73.32	1.746937	12.35271	69.8094	76.8306
Control	42	73.02381	1.765421	11.44123	69.45847	76.58915
Combined	92	73.18478	1.238702	11.88122	70.72425	75.64531
diff		0.2961905	2.500407	-4.671305	5.263686	

*P* = 0.55, *t* = 0.1185, degrees of freedom = 90; based on the above *P* value of 0.55, there is no significant difference in performance of students exposed to human skull with the ones exposed to wooden skull in the overall exam. Note that the confidence intervals also overlap while the mean is also the same. Two-sample *t*-test with equal variances.

**Table 5:** 

Total	Active group	Control group	Total
40-50%	2	1	3
51-60%	9	6	15
61-70%	8	11	19
71-80%	17	14	31
81-100%	14	10	24
Total	50	42	92

Pearson chi^2^ (4) = 1.6811; Pr = 0.794; basing on the above *P* value of 0.79, there is no significant difference in performance of students exposed to human skull with the ones exposed to wooden skull in all categories. Because of the small number of failures in the two groups, we used Fisher's exact test to determine the difference in this category.

**Table 6:** 

Total	Active group	Control group	Total
40-50%	2	1	3
51-60%	9	6	15
61-70%	8	11	19
71-80%	17	14	31
81-100%	14	10	24
Total	50	42	92

Fisher′s exact = 0.807; based on the above *P* value of 0.81, there is no significant difference in failure of students exposed to human skull with the ones exposed to wooden skull.

## Data Availability

The metadata used to support the findings of this study are available from the corresponding author upon request.
